# Group interpersonal psychotherapy for generalized anxiety disorder: development process and a pilot test

**DOI:** 10.1016/j.clinsp.2025.100664

**Published:** 2025-04-24

**Authors:** Xia Sun, Lujia Zhang, Yan Pan, Kaiji Ni, Chenfeng Ji, Qian Zhou, Scott Stuart, Yanli Luo

**Affiliations:** a Department of Psychological Medicine, Renji Hospital, School of Medicine, Shanghai Jiao Tong University, Shanghai, PR China; b Guanxin Shanghai Tongchuang Future Psychiatric Clinic Co., Ltd, Shanghai, PR China; c Department of Psychiatry, University of Southern California, Los Angeles, CA, USA; d IPT Institute, Coralville, IA, USA

**Keywords:** Group interpersonal psychotherapy, Generalized anxiety disorder, Development, Adaptation, Mental health, Suicide, Interpersonal Relationships, Quality of Life

## Abstract

**Background:**

Generalized Anxiety Disorder (GAD) is a common and disabling psychiatric disorder that negatively impairs patients' quality of life. Studies suggest that inadequate consideration of interpersonal problems may contribute to the poor treatment response in psychotherapy for GAD. Interpersonal Psychotherapy (IPT) is an evidence-based psychosocial intervention that focuses on symptoms by improving interpersonal functioning, and research demonstrates that IPT is efficacious in treating social anxiety and panic disorders. To date, however, there are no studies examining IPT for patients with GAD.

**Methods:**

The authors developed and pilot-tested an IPT manual for the treatment of GAD in groups with two cohorts of patients. Pre- and post-test measures of anxiety were collected and analyzed.

**Results:**

The authors successfully developed a treatment manual for Group IPT for Anxiety using iterative qualitative methods. Significant improvement from baseline was noted in the GAD scores for patients in both patient cohorts.

**Conclusions:**

The results suggest that IPT can be successfully used for GAD in group formats and that future large-scale randomized trials should be warranted. The results also suggest that online group therapy is feasible and warrants further study and that it may be used to deal with access and logistical barriers faced by many patients.

## Highlights

• A novel Group IPT was developed for Generalized Anxiety Disorder (GAD).
• Reduction in GAD symptoms highlights the potential efficacy of Group IPT for GAD.
• N Group IPT effectively addresses mental health care access barriers.

## Background

Generalized Anxiety Disorder (GAD) is a common, chronic, and disabling mental disorder characterized by persistent excessive anxiety and uncontrollable worry.^1^^,^^2^ GAD typically begins in adulthood and persists over time, affecting 4 %–7 % of the population during their lifetime worldwide.^3^ A meta-analysis showed that in China the current and lifetime prevalence of GAD was 5.17 % (95 % CI 3.72 %–6.63 %) and 4.66 % (95 % CI 3.17–6.14) respectively.^4^ The excessive daily worries and chronic course severely impair patients' quality of life, interpersonal relationships, social and occupational functioning, and increase economic burdens on individuals, families, and society globally.^5^ Suicide risk is also increased with anxiety disorders, further adding to the importance of treatment.^6^^,^^7^

Despite the high morbidity, treatment rates for GAD are quite low, especially in middle-income (29.1 %) or low-income (21.7 %) countries.^3^ Although clinicians as well as patients prefer treatment with psychotherapy as opposed to medication, nearly half of GAD patients who receive psychotherapy do not show a reliable reduction in anxiety symptoms.^8^ Many studies have provided support for the proposition that inadequate consideration of interpersonal problems may contribute to the poor treatment response in psychotherapy for GAD.^9^^,^^10^ Cognitive Behavioral Therapy (CBT), while a first-line empirically-supported psychotherapy for GAD, does not focus specifically on interpersonal issues.^11^ Therefore, in order to improve treatment outcomes for GAD, it is crucial to consider how to optimize treatment approaches to address interpersonal concerns.

Interpersonal Psychotherapy (IPT) is a time-limited, structured and evidence-based treatment that focuses on relieving symptoms by improving interpersonal functioning.^12^, ^13^, ^14^ IPT was first developed by Klerman and colleagues as a treatment for unipolar depression,^13^ and has since been conceptualized as a transdiagnostic treatment that is grounded in Interpersonal Theory and Attachment Theory. IPT links the onset and maintenance of psychological distress to life events, especially in three interpersonal problem areas of grief and loss, interpersonal disputes, and role transitions.^14^

After decades of studies, IPT has been successfully adapted for a variety of clinical disorders, including eating disorders,^15^ PTSD,^16^ and others.^17^^,^^18^ IPT has also been delivered for individuals, groups,^19^ families,^20^ and couples,^21^ as well as via video and telephone.^22^^,^^23^ Research on Social Anxiety Disorder (SAD) and Panic Disorder (PD), which are closely connected to GAD, suggests that IPT effectively reduces patients' anxiety symptoms.^24^, ^25^, ^26^ However, there are not yet IPT studies for GAD in either individual or group modalities.

In contrast to the theoretical approach underlying CBT (e.g., change in cognitions which then impacts anxiety), IPT for anxiety disorders focuses on the interpersonal context of the anxiety and is designed to help patients request the support and reassurance they need, and to get their interpersonal and attachment needs better met during times of crises. This in turn leads to a reduction in symptoms. The key elements of IPT include increasing social support and improving interpersonal functioning, as well as teaching patients to better communicate their anxiety and distress in ways that others can respond to with reassurance.^14^

Moreover, the group treatment is designed to provide immediate social support from others who can understand the patient's distress, another hypothesized mediator in IPT. The group provides initial support as the patient works to increase social support outside of the group as well.

The authors undertook this pilot study with two aims: 1) Developing and adapting a manualized Handbook for Group IPT for GAD in China; and 2) Concurrent testing of the manual in an open trial with several groups.

## Methods

### Development of the group IPT for GAD manual

The authors developed the initial group-IPT treatment handbook for Chinese GAD patients by reviewing the literature on both GAD and IPT generally, as well as IPT for Groups. The authors also reviewed treatment manuals including Interpersonal Psychotherapy: A Clinician's Guide (second edition),^14^ Group Interpersonal Therapy (IPT) for Depression^27^^,^^28^ as well as the IPT for Groups Psychotherapy Handbook.^29^ The initial pilot group was primarily based on a modification of the IPT for Groups Handbook modified for anxiety.

The authors then conducted pilot group one, with iterative changes in the manual made after concluding that group. The same process was used for pilot group two, which resulted in the final treatment manual. Both groups were included in the overall sample used to analyze the outcome.

Participants in this study were part of a larger treatment trial who were allocated to receive either Group IPT and sertraline for GAD or sertraline and psychoeducation. Only participants who received Group IPT were included in the manual development.

The two pilot IPT groups were conducted from September 2021 to December 2022. Each group consisted of eleven patients and two certified IPT Community-Based therapists who were supervised by a certified IPT supervisor. All group sessions were conducted online with video.

Participants were recruited via outpatient clinics, recruiting posters, and WeChat official account advertising in the hospital. Patients between the ages of 18 to 65 were eligible if they were: 1) Diagnosed with GAD according to DSM-5 criteria^30^;; 2) Were experiencing their first episode of GAD; 3) Had not been treated with antidepressant or antianxiety medication; 4) Had not received IPT. Exclusion criteria included: 1) Any mental illness diagnosis other than GAD; 2) Suicidality; 3) Current or previous history of alcohol or drug addiction.

## Results

### Quantitative data

**Fig. 1:**
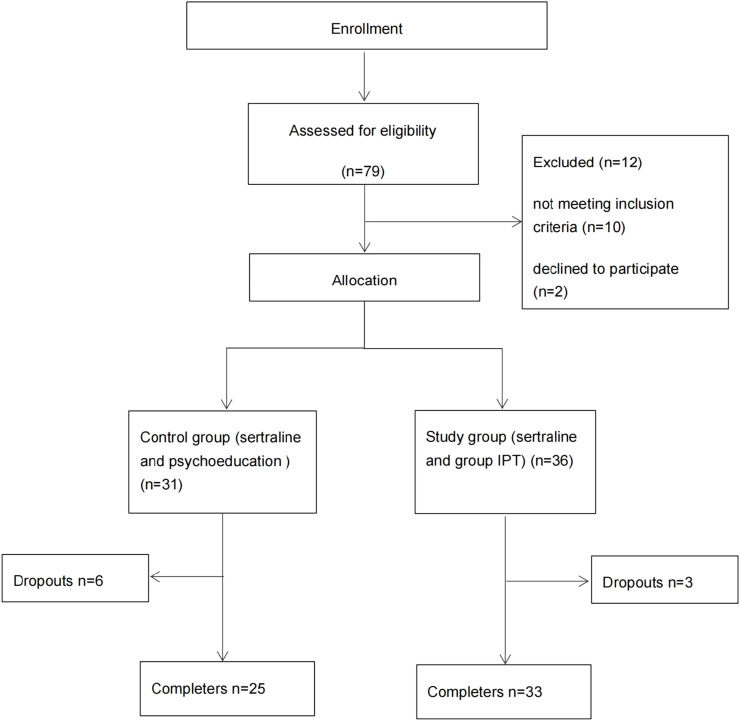
Consort flow‐chart of participants’ allocation to study.

A total of 79 subjects were screened for the larger study. Fig. 1 provides a consort diagram of participant flow (Fig. 1). Twelve were excluded because they did not meet inclusion criteria (*n* = 10). Two declined to participate. No symptomatic data is available for these subjects.

The remaining 67 subjects were randomized to receive either sertraline and psychoeducation (*n* = 31) or sertraline and group IPT (*n* = 36). Psychoeducation was restricted to providing information about the treatment and prognosis of GAD, including medication use and side effects. All subjects were treated with sertraline (50‒150 mg daily). Anxiety and depression symptoms were measured using the 7-item Generalized Anxiety Disorder Questionnaire (GAD-7) at baseline, week-1, week-2, week-4 and week-8 (post-treatment).

The dropout rates were 8.33 % in the sertraline plus group IPT versus 19.36 % for the sertraline plus psychoeducation group (λ^2^ = 0.921, *p* = 0.337, see Table 1). Wilcoxon rank sum test for two paired samples and Mann Whitney u test for two independent samples were conducted to compare GAD scores within and between two groups. Compared with week-0 (intake), both groups showed a significant decrease in GAD score in week-8 (Sertraline Plus Group IPT, *Z* = 4.786, *p* < 0.005; Sertraline Plus Psychoeducation, *Z* = 4.291, *p* < 0.005) (see Table 2). There was no significant difference between the groups in Week-0 (*U* = 0.417, *p* = 0.677) and Week-8 (*U* = 0.2, *p* = 0.841) (see Table 2).

**Table 1:** Results of chi-square test of participation of two group.

	Feature	Sertraline Plus Group IPT (*n* = 36)	Sertraline Plus Psychoeducation (*n* = 31)	λ^2 a^	p
**Participation**	Complete	33 (91.67 %)	25 (80.64 %)	0.921	0.337
	Drop out	3 (8.33 %)	6 (19.36 %)		

Notes: ^a^Used Chi-Square test.

**Table 2:** Comparison of GAD scores within and between two groups.

GAD-7	Sertraline Plus Group IPT (*n* = 33)	Sertraline Plus Psychoeducation (*n* = 25)	*U*^b^	p
Week-0	14.06 (5.123)	13.60 (4.761)	0.417	0.677
Week-8	3.91 (2.944)	3.96 (3.169)	0.200	0.841
*Z* value^a^	4.786	4.291		
p	0.000^c^	0.000^c^		

Notes: ^a^ Used Wilcoxon Analysis.

b Used Mann Whitney *U* test.

c *p* < 0.05. GAD-7, The 7-item Generalized Anxiety Disorder Questionnaire.

Between group comparisons (Sertraline Plus Group IPT vs. Sertraline Plus Psychoeducation) using two-way ANOVA (week-0, week-4, and week-8) showed that there were significant Time differences (*F* = 159.457, *p* < 0.005), indicating that GAD-7 scores changed over time without considering Intervention factors (see Table 3). There were no significant differences in the Intervention main effect and Intervention × Time interaction effect.

**Table 3:** Results of two-way ANOVA for Intervention, time and intervention×time on GAD-7 Score between groups.

	Time			Two-Way ANOVA		
	Week-0 (*M*±SD)	Week-4 (*M*±SD)	Week-8 (*M*±SD)	F	p	Partial (η^2^)
Sertraline Plus Group IPT	14.06 ± 5.123	4.66 ± 3.730	3.91 ± 2.944			
Sertraline Plus Psychoeducation	13.60 ± 4.761	4.67 ± 3.212	3.96 ± 3.169			
Intervention main effect				0.002	0.962	0.000
Time main effect				159.457	0.000^a^	0.796
Intervention × time interaction effect				0.020	0.960	0.000

Notes: ^a^*p* < 0.05.

GAD-7, The 7-item Generalized Anxiety Disorder Questionnaire.

### Qualitative data

As described in the IPT for Groups Handbook^29^ the initial pilot groups included two 50-min pre-group individual intake meetings and eight 90-minute group sessions organized into initial, intermediate and termination phases. The primary goal was to decrease anxiety symptoms by: 1) Providing immediate social support within the group; 2) Resolving interpersonal distress in the areas of disputes, role transitions, and grief and loss; 3) Improving interpersonal functioning generally; and 4) Enhancing patient's social support outside of the group. Table 4 describes the aims and agendas for each session.

**Table 4:** Aims and agenda for Group IPT for GAD sessions (see Stuart and Schultz, 2016).

Phase	Sessions/Meetings	Aims	Agenda
Pre-group	Meeting 1	Determine if the patient is suitable for group	Conduct Psychiatric Evaluation
			Conduct Interpersonal Inventory
			Assess Suitability for Group IPT
			If not suitable, refer to individual therapy
	Meeting 2	Explain how group IPT works	Develop Interpersonal Summary & Formulation
			Relate anxiety to interpersonal problem areas
			Explain how Group IPT works
Initial	Session 1	Create a warm and support group atmosphere	1. Warm up
			2. Ice breaker: introduce group member to each other
		Introduce Group IPT purpose and structure	3. Introduce purpose and structure of Group IPT
			4. Discuss group rules
		Emphasize commonality and give hope	5. Identify individual and group goals
			6. Assign homework
	Session 2	Create a warm and support group atmosphere	Brief check in and review homework
		Share successful coping strategies for anxiety	Discuss personal successful coping strategies for anxiety
		Link anxiety and interpersonal problem areas	Share Interpersonal Inventory
			Assign homework
Intermediate	Session 3	Focus on Interpersonal Area: Role Transition	Brief check in and review homework
			Introduce Role Transition and Tool of Timeline
		Emphasize commonality and provide support	Draw Timeline and exercise in subgroups
			Demonstrate and share individual Timeline to Group
			Assign homework
	Session 4	Address Interpersonal Area: positive impact of Role Transition	Brief check in and review homework
			Discuss positive impact of Role Transition in subgroups
		Emphasize commonality and provide support	Briefly share individual experience to Group
			Assign homework
	Session 5	Focus on Interpersonal Area: Interpersonal Conflict	Brief check in and review homework
			Introduce Interpersonal Conflict and Tool of Interpersonal Ladders
		Emphasize commonality and provide support	Draw Interpersonal Ladder and exercise in subgroups
			Demonstrate and share individual Ladder to Group
			Assign homework
	Session 6	Address on Interpersonal Area: Problem Solving Strategy	Brief check in and review homework
			Introduce Problem Solving Strategy
		Emphasize commonality and provide support	Discuss and exercise Problem Solving Strategy in subgroups
			Briefly share individual experience to Group
			Assign homework
	Session 7	Focus on Interpersonal Area: Grief and Loss	Brief check in and review homework
			Introduce Grief and Loss and Tool of Timeline
		Emphasize commonality and provide support	Discuss and mourn losses in subgroups
			Briefly share individual experience to Group
			Assign homework
Termination	Session 8	Review Group IPT progress	Brief check in
		Discuss future treatment if needed	Review Group IPT progress, especially goals and accomplishments
		Say goodbye	Discuss rules of Wechat group
			Send blessings and best wishes to group members (Homework of session 7)
			Say goodbye

### Development of group IPT for GAD manual

Pre-Group Intake (individual meetings)

The pre-group individual meetings had two main goals: to determine if the patient was suitable for group treatment and to explain how Group IPT works. The therapist conducted a psychiatric evaluation, reviewed symptoms, and confirmed the GAD diagnosis. An Interpersonal Inventory was constructed,^14^ and relationship problems were linked to anxiety symptoms. The therapist and patient also collaboratively developed a written IPT Summary^12^^,^^14^ noting the issues that led to the current distress, listing the patient's strengths and resources, and setting goals for the group work. This written Summary was also used by patients as an outline to introduce themselves in the first group session.

Group therapy: initial phase (group sessions 1‒2)

The main goals of the initial phase of Group IPT were to create a warm and supportive group atmosphere, introduce Group IPT's purpose and structure, and link anxiety with the interpersonal problem areas. For the online Group IPT, all members were required to keep their video cameras on to promote participation.

Qualitatively, the authors discovered that some patients with GAD were anxious about sharing their experiences or ideas in a group, particularly online, so the authors developed a series of subgroup therapy activities as part of the initial phase. Patients first shared their experiences in these subgroups, then shared in the larger group.

For example, in session 1, after the therapists modeled introducing their co-therapist, patients were placed into subgroup pairs and asked to introduce themselves to their partners. They were given about 10 min within their subgroups and then returned to the full group where they introduced their partner to the other group members.

The authors also discovered that, in contrast to classic psychodynamic groups in which patients are told they cannot have any contact outside of the group, the patients became more engaged and benefitted from the establishment of a WeChat social media group in which members submitted homework and reviewed homework from others, commented on it, and provided support to one another between groups. This reduced participation anxiety, built social support, and encouraged engagement. The authors note the use of WeChat is ubiquitous in China and is a culturally relevant way of adapting IPT.

#### Intermediate group phase (group sessions 3‒7)

The main goals of the intermediate phase of Group IPT were to address the interpersonal problem areas of Role Transition, Interpersonal Disputes, and Grief and Loss. The authors designed the groups to sequentially address the IPT problem areas, starting with role transitions, then disputes, and finally grief and loss. The authors believed that doing so would provide gradual exposure to more difficult topics as the group progressed and coalesced. This appeared to be the case. As the groups became safer and more supportive, progressively deeper topics were discussed. The authors also discovered that the written tools (such as the Timeline and Dispute Graph) developed by Stuart and Robertson^14^ were particularly helpful for online group therapy, as they provided a structure that patients could utilize between sessions. There are many members who remarked that after using the Dispute Graph, it would immediately appear in their minds with subsequent disputes, and they would utilize the concepts to evaluate the seriousness of the conflict and the importance of the relationship, which was vital for managing their emotions and maintaining relationships. The tools also facilitated practicing interpersonal communication skills outside of the group.

#### Termination of group phase (group session 8)

The main goals of the termination phase of Group IPT were to review progress, emphasize therapeutic gains, and say farewell. Members were encouraged to express their thoughts and feelings about formally ending the group.

The authors discovered that patients in the groups both wanted and benefitted from continued support from one another utilizing the Wechat group after the group therapy was formally terminated. Accordingly, the authors collaboratively developed rules for the WeChat group in the last session. The group leaders also participated in the Wechat group during the first three months after termination to continue to provide support when needed.

### Qualitative data and subsequent adaptations to group IPT for GAD

In general, participants liked the group format and considered some IPT tools to be useful to them. Representative comments are summarized in Table 5.

**Table 5:** Qualitative feedback from Group IPT participants.

Participant	Favorite part	Most helpful part
Participant 1	The frank, open, support and warm atmosphere in group. I don't feel so lonely.	“The Timeline ‒ I can talk about my problems”.
Participant 2	Group members gather together to talk about anxiety. “It (the group) reduced my loneliness”.	Courage; a sense of security.
Participant 3	Communication with group members.	I achieved my goals, and I was touched.
	Empathy from others.	
Participant 4	It (the group) was a platform and an opportunity to share and communicate with group members.	I realize that anxiety is a common phenomenon.
	The Group IPT is like an output catalyst.	Treat anxiety effectively.
Participant 5	Team members have a lot in common.	Hearing other group members’ methods to deal with anxiety.
	We can talk and listen.	The treatment process of discussing anxiety with each other.
Participant 6	It (the group) was a place to pour out my worries and upset.	The leaders' feedback, such as more participation in activities/change myself/trying new things.
		Recommended books.
		Tools such as interpersonal ladders.
Participant 7	“Listening to others” was relaxing.	The Timeline was a systematic analysis of myself.
		All aspects (of my anxiety) have improved.
Participant 8	Hearing common experiences.	It helped with more relaxation.
	It's better to listen to others' ideas than dwelling on my own.	I am more receptive to myself.
	It (the group) stretched my mind	It helped with more relaxation.
Participant 9	Listening to others’ stories.	Interpersonal ladders helped with problem solving.
		I am more relaxed, not so nervous.
Participant 10	Hearing that other people also have anxiety symptoms.	The IPT written tools.
	Learning that anxiety is treatable.	Exchanging members' coping methods for anxiety and summarizing my own methods.
	Hope for treatment.	
Participant 11	Being with group members who have the same problems.	Group members have many useful tricks which are very useful when needed.
	Developing friends and support.	Know many friends with similar problems. Before, we were isolated islands, now others accepted, tolerated, and connected us.
	Sharing some experiences and ideas.	

The authors collected qualitative data from several sources. First, before each session, the authors used ongoing supervision to review the content of the previous session and emphasized key points of the next session with a therapy-to-supervision session ratio of 1:1. The two therapists reported in detail each group member's feedback on each agenda and discussed it with supervisors. Second, during the last session of each group, the therapists discussed with the group members the most helpful parts of the therapy and the parts that they thought needed to be adjusted based on the patient's experiences. Therapists asked these questions directly and told group members their feedback would help to improve the treatment program, while group members talked one by one. The least helpful parts and least useful parts are parts that they thought needed to be adjusted. Third, the authors collected information from the therapists' and supervisor's observations about what did and did not work well in the group. The final protocol was the product of this iterative qualitative approach.

Based on this data from the two pilot groups, the authors made a number of adaptations to the final protocol. Primary among those was the creation and continued use of a WeChat group including all members of the IPT group. To further utilize this as an engagement tool, the authors discovered that it was helpful for group leaders to share therapy notes after each session in the WeChat group, and found it was effective to have group members submit their homework through WeChat. Group leaders managed the Wechat group during the treatment and transferred it to a delegated group member in the last session, but the leaders actively participated in the Wechat group for three more months to provide maintenance support. Having a WeChat group appeared to be a great continuation of social support and was strongly endorsed by the group members, therapists, and supervisor.

Second, the authors discovered that it was helpful to pay more attention to treatment goals rather than the patient's symptoms. Focusing too much on anxiety symptoms appeared to aggravate some patients’ worries. In contrast, focusing on goals and social functioning provided patients with a sense of hope and promoted positive changes.

Third, the authors discovered that it was extremely helpful to assign specific homework between each session. The most successful in terms of participation was assigning patients the task of taking photos of themselves engaging in social activities. Compliance was nearly universal and fostered the IPT goal of engaging more social support. Patients enjoyed showing the pictures and sharing stories about them in the group, and the authors discovered that they also shared the photos and brief descriptions in the WeChat group between sessions as well.

Near the end of the first group, the authors began assigning this photo homework for each session, then continued it in the second group. To ensure confidentiality and privacy, patients could choose certain pictures to “represent” what they wanted to convey, such as a male patient using a picture of “pepper” to represent a conflict with his wife over pepper, or a patient using a picture of a park to represent a weekend of playing at the park with his family. These were then discussed in the therapy sessions. Typical homework is shown in Table 6.

**Table 6:** Typical homework for Group IPT for GAD.

Sessions	Theme	Homework
Session 1	Create a warm and support group atmosphere	Share a photo of a caring relationship that brings warmth
Session 2	Interpersonal Inventory	Share a photo and record an activity with person in middle or inner circle of interpersonal inventory
Session 3	Interpersonal Area: Role Transition	Share a photo of Timeline event to commemorate Role Transition
Session 4	Positive Impact of Role Transition	Complete a sentence (before change, I…; now I …; in the future, I will …; if you want to understand me, you need to know…)
Session 5	Interpersonal Area: Interpersonal Conflict	Share a photo represents an example of a past conflict
Session 6	Problem Solving Strategy to deal with Interpersonal Conflict	Share one or more tips for solving interpersonal conflicts
Session 7	Interpersonal Area: Grief and Loss	Think of a name for Wechat group.
		Prepare something positive to say farewell to other group members and share in last session
Session 8	Ending Group therapy	/

Fourth, the authors consistently used PowerPoint presentations to review the agenda, main contents, and homework assignments for each online session. Sharing and synchronous selected typing of group members’ feedback on the shared online screen improved cohesion and helped make group members feel valued. In addition, group leaders shared PowerPoint notes in the WeChat group which members could review. Online therapy allowed us to use technology like this to our advantage.

## Discussion

This project was undertaken to develop a Group IPT manual for intervention for patients with generalized anxiety disorder in China and to collect preliminary data supporting its clinical utility. To the best of our knowledge, this is the first study to evaluate Group IPT for treatment of GAD, it is also one of the first IPT outcome studies conducted in China. Based on the data the authors collected, we now have an operationalized manual for Group IPT for GAD.

There are several important factors to note. First, the Group IPT approach generally and the approach specifically differs from “classic” psychotherapy groups, such as those described by Yalom & Leszcz.^31^^,^^32^ For instance, the IPT groups are very short, specifically directed towards social support, and very structured. They are not designed to change personality or attachment style, but to foster improved communication and social support to improve functioning and decrease symptoms rapidly. The IPT goal of increasing social support is achieved by utilizing group members to support one another as quickly as possible.^14^^,^^29^

Dropout is an important indicator of treatment acceptability and client engagement.^33^ In comparison to the Sertraline Plus Psychoeducation group, the low dropout rate of Sertraline Plus Group IPT (8.33 % vs. 19.36 %) in the current study provides evidence suggesting that GIPT may as a more accepted treatment for GAD patients. Some studies have investigated the treatment dropout rate for this population. Malivoire BL et al.^34^ found the drop-out rate of Group CBT (12 sessions) for GAD patients was 15.3 % (*n* = 50), while in another group CBT for GAD study, the drop-out rate was 30 % (*n* = 37).^35^ Based on systematic reviews and meta-analyses, Elon Gersh et al.^33^ calculated the weighted mean dropout rate for GAD in individual psychotherapy was 16.99 %, while 17.14 % in Cognitive Behavioral Therapy and 16.57 % for Psychodynamic Therapy. Furthermore, in a previous meta-analysis research examined IPT dropout rates for mental health disorders in randomized controlled trials, Jake Linardon et al.^36^ found the weighted mean dropout rate from IPT was 20.6 %, 16.1 % for anxiety disorders, and 13.8 % for group face-to-face format. Obviously, the present study has a much lower dropout rate in Group IPT, which the authors attribute to thte constant adaptation during the manual development process based on the Chinese population and culture. Adaptations specific to China included the use of social media (WeChat specifically) to engage group members and to foster social activities between sessions. This led to increased group participation and cohesion and may have decreased drop-out rates. Concerns about confidentiality and privacy on Wechat media will need to be addressed in the future, though the authors did not have any patients raise concerns about those issues during treatment. Moreover, the authors discovered that at least in China, patients pushed very hard to continue to communicate with one another after group termination. The Wechat groups continued after the formal treatment was completed and appeared to provide support for patients. The authors noticed that some members would post meditation music or articles related to mental health in the group, while some members also shared news about marriages, births, clinic visits, etc. Overall, the existence of the WeChat group was also a transition for group members, protecting the support of the group rather than coming to an abrupt end. The acceptability and impact of using social media in this way after the formal termination of therapy groups will need to be explored in other cultural settings.

In this present study, both groups showed a significant decrease in GAD score in week-8 (Sertraline Plus Group IPT, *Z* = 4.786, *p* < 0.005; Sertraline Plus Psychoeducation, *Z* = 4.291, *p* < 0.005), but no significant differences were found between groups (*U* = 0.2, *p* = 0.841) at post-treatment. The result is consistent with the study that investigated the effectiveness difference between Acceptance‐Based group Behavioral Therapy (ABBT) and standard nondirective supportive group therapy (NDST) for GAD patients, which also showed a significant effect for Time but not for the Treatment main effect.^37^ As CBT is considered the “golden standard” and first-line psychotherapy for GAD, Simona Stefan et al.^38^ conducted a randomized clinical trial to compare three CBT protocols for GAD: a) Cognitive Therapy/Borkovec's treatment package, b) Rational Emotive Behavior Therapy, and c) Acceptance and Commitment Therapy/Acceptance-based behavioral therapy, results suggested that all three approaches appear to be similarly effective. Naomi MS et al.^39^ compared group CBT, Yoga, and Stress Education for GAD patients who reported Group CBT was more effective than Yoga and Stree Education. Since the research is a preliminary exploration, more studies are worth investigating in the future especially a comparison between GCBT and GIPT.

Clearly, there were strong and supportive interpersonal connections developed within the group, a factor that is hypothesized to be a powerful mediator in IPT. This may have also helped patients develop a sense of hope and countered the demoralization often experienced by patients with anxiety and other mental health issues. Demoralization, which will be introduced as a formal concept in the next iteration of the DSM, also appears to be linked to suicide risk.^40^ Unfortunately, there are no linkage studies to date connecting improvement in interpersonal issues to improvement in anxiety; however, the present study certainly suggests they are warranted.

There are some limitations. First, this is a lack of a control comparison in this study. The authors only compared the group differences between Sertraline Plus Psychoeducation and Sertraline Plus Group IPT, a Sertraline monotherapy group or an IPT monotherapy group would have provided more reference for the efficiency of each treatment. Second, this study did not include follow-up assessments, which limits our long-term observations of treatment effects. Third, the authors lack additional symptomatic and interpersonal measures to better understand the connection between interpersonal issues addressed in therapy and improvement. Although the authors emphasized the importance of interpersonal relationships in GAD treatment and hypothesized that GIPT improves anxiety symptoms by providing social support, there was no assessment of interpersonal relationships during the research process. Last but not least, patients were carefully selected for the absence of comorbidity, and the qualitative data obtained are relevant only to patients in China. Thus, these findings may not extend to other clinical settings. In summary, this current study provides a small first step in highlighting the acceptable and feasible GIPT for GAD patients in China. Future research could use community-based clinical samples and continue to investigate more deeper to enhance the understanding of GIPT for GAD patients.

## Conclusions

The authors are positive about the scalability of Group IPT for GAD and the promise of increased access. Online treatment, particularly in China, appeared to be highly acceptable and feasible and can allow patients who would otherwise be unable to access treatment to participate. This is particularly relevant for rural areas, but also for dense urban areas in which travel time via public transportation is also a barrier.

## Conflicts of interest

The authors declare no conflicts of interest.
